# Tekt3 Safeguards Proper Functions and Morphology of Neuromast Hair Bundles

**DOI:** 10.3390/ijms26073115

**Published:** 2025-03-28

**Authors:** Dongmei Su, Sirun Lu, Ling Zheng, Dong Liu

**Affiliations:** 1 Department of Neuroscience, School of Life Sciences, Shenzhen 518055, China; 12131314@mail.sustech.edu.cn (D.S.); 12211253@mail.sustech.edu.cn (S.L.); 2 Department of Biomedical Engineering, Southern University of Science and Technology, Shenzhen 518055, China; 11849591@mail.sustech.edu.cn

**Keywords:** zebrafish, tekt3, hair cell, cilia, regeneration

## Abstract

The inner ear and/or lateral line are responsible for hearing and balance of vertebrate. The otic sensory hair cells (HCs) employ cilium organelles, namely stereocilia and/or kinocilia, to mediate mechanical stimuli to electrical signal transition. Tektins (Tekts) are known as the cilium microtubule stabilizer and inner-space filler, and four Tekt(1-4)-encoding genes are identified in zebrafish HCs, but the subcellular location of Tekts in HCs remains unknown. In the present study, we first found that *tekt3* is expressed in the inner ear and lateral line neuromast. Antibody staining revealed that Tekt3 is present in neuromast and utricular HCs. It is absent in the saccule, the authentic hearing end-organ of zebrafish and the crista of semi-circular canals. Furthermore, Tekt3 were enriched at the apical side of neuromast and utricular HCs, mainly in the cytosol. Similar subcellular distribution of Tekt3 was also evident in the outer HCs of mature mouse cochlea, which are not directly linked to the hearing sense. However, only neuromast HCs exerted morphological defect of kinocilia in *tekt3* mutant. The disrupted or distorted HC kinocilia of mutant neuromast ultimately resulted in slower vital dye intake, delayed HC regeneration after neomycin treatment, and reduced startle response to vibration stimulation. All functional defects of *tekt3* mutant were largely rescued by wild-type *tekt3* mRNA. Our study thus suggests that zebrafish Tekt3 maintains the integrity and function of neuromast kinocilia to against surrounding and persistent low-frequency noises, perhaps via the intracellular distribution of Tekt3. Nevertheless, TEKT3/Tekt3 could be used to clarify HC sub-types in both zebrafish and mice, to highlight the non-hearing HCs.

## 1. Introduction

Vertebrates’ sense of hearing and balance rely on the function of mechanosensory receptors (hair cells, HCs) in the inner ear and/or lateral line. HCs employ the actin-filled stereocilia and/or kinocilia to detect physical stimuli such as soundwaves and vibration and transduce them into nerve signals via opening mechanoelectrical transducer (MET) channels. HC kinocilium is a single non-motile cilium, also called the true cilium. The kinocilia are different from non-motile primary cilia and motile cilia/flagella in that each kinocilium possesses the 9+2 axonemal MTs yet it is non-motile, due to a lack of inner dynein arms and nexin links [1,2]. Among vertebrate HCs, there is a great diversity in kinocilial morphology—from the transient kinocilia of cochlear HCs, the bulbed kinocilia of amphibian HCs, to the towering kinocilia of lateral line neuromasts [2,3]. However, little is known about the molecular bases of this morphological diversity and if functional diversity of these different shaped kinocilia also exists. Furthermore, since the mature vestibular HCs of all vertebrates possess both kinocilia and stereocilia, while mature cochlear HCs do not have any kinocilia, the roles of kinocilia played in the cochlear vs. vestibular HC differentiation and during the gain of hearing warrant in depth exploration.

Tektins (Tekts) are a highly conserved family of coiled-coil domain-containing proteins. They are known to play a role in the structure, stability, and function of cilia and flagella [4]. Tekts form filaments to run the length of the axoneme along the inner surface of the A tubule of each ‘9+2’ microtubule (MT) doublet. For motile cilia and flagella of both unicellular and multicellular organisms, enabling the movement of axoneme is one of the common features of Tekts. The members of Tekt family, ranging from one to up to five in each species, are all derived from an ancestral Tekt member. Evolutionarily, tektin1, 2 and 4 encoding genes are usually co-regulated to show similar spatial and temporal expression patterns. The expression of Tekt3 or 5 encoding (3/5) gene appears in species-specific cell types including the sensory cells. For instance, sea urchin tektin filaments are composed of hetero- and homodimers of Tekt1, 2 and 4. Recent structural analyses reveal a complex arrangement of filaments made of all tektins, including Tekt3/5 in bovine respiratory cilia and mammalian and sea urchin sperm flagella [5,6,7,8]. In general, Tekt1, 2 and 4 appear to form a conserved core of filaments while species-specific Tekt3/5 are added depending on species and cell types. Furthermore, the presence or absence of Tekt3/5 is likely a key factor in differentiating cilia type and function, between sensory cell (with multiciliary arrays) and motile cilia of the other ciliated structures. The complex composition and decoration of tektin filaments within the axoneme may contribute to cilia diversity and function, most likely by fine-tuning the stability of the axoneme to withstand mechanical and beating forces. However, little is known about the role of tektins, particularly the Tekt3 that is specifically expressed in zebrafish HCs [9,10,11] and associated with nodal and olfactory cilia in functional knockdown experiment [12].

Previous studies indicate that mutations of individual tektins lead to structural and functional defects of cilia and flagella in green algae, zebrafish, rodents, and humans [13,14,15,16,17,18,19,20]. In humans, all tektins, including TEKT3, are found in the spermatozoa, mainly localized to the flagella, and present in the respiratory doublet microtubules. In rodent species, TEKT3 is found in the sperm/spermatozoa flagella, head, and acrosome membrane, and *Tekt3* KO mouse sperms exert reduced motility and forward progression and increased flagellar bending. Point mutations in patients with a complete loss of *TEKT3* expression causes the acrosomal hypoplasia with no apparent defects in sperm flagellar but decreased progressive motility of their sperms [14]. However, all these studies focus on sperm and male infertility, whether these mammalian mutants have any hearing and vestibular defects is yet to be reported.

The zebrafish genome contains four tektin-coding genes (1–4), and these genes, from *tekt1* to *tekt4*, are expressed in otic and/or neuromast HCs [9,10,11,21]. Interestingly, we have found that during HC development and regeneration of larval zebrafish, only *tekt3* expression was kept in mature HCs. To understand the role of *tekt3* in HC development and function, the present study obtained a loss-of-function zebrafish mutant, *via* CRISPER/Cas9 technique, that developed normally and was fertile. Tekt3 was inclusively expressed in utricular and neuromast, present and enriched in the cytosol at the apical end of HCs. Such a TEKT3 distribution was also found in the outer HCs of mouse cochlea. However, only neuromast HCs of *tekt3* mutant exerted clustered, bent and/or bubbled kinocilia. Functional defects included vital dye intake, delayed HC regeneration after neomycin was used to kill lateral line HCs, and reduced startle response to vibration stimulation. On the other hand, the normal VOR score of *tekt3* mutant indicated that utricular HC development and function was not affected, suggesting that zebrafish Tekt3 is involved in a protective mechanism to enable structural stability of HC kinocilia that are constantly in action.

## 2. Results

### 2.1. Zebrafish Tekt3 Is Specifically Expressed in the Ear and Neuromast

**Figure 1:**
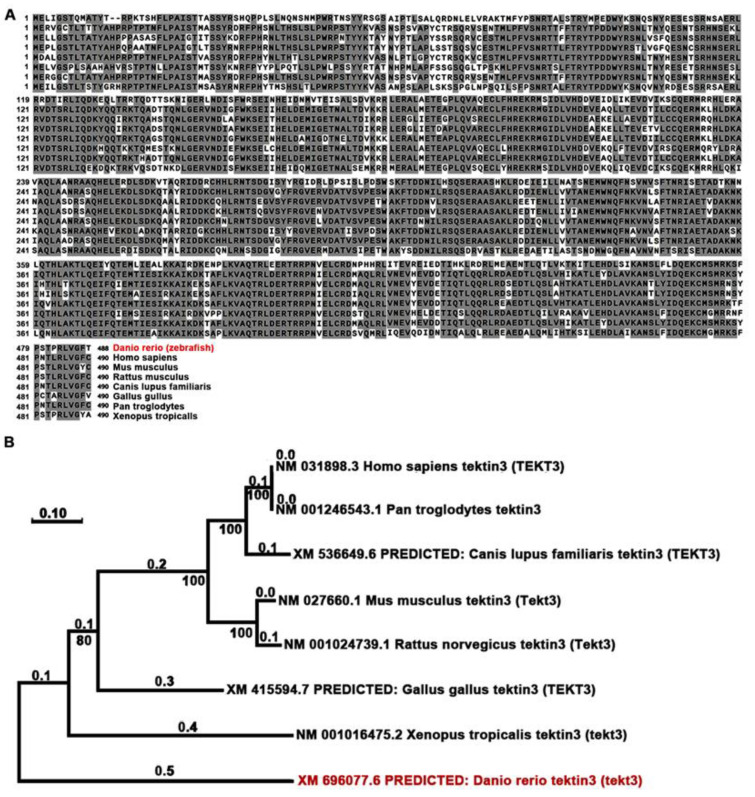
TEKT3/Tekt3 is evolutionarily conserved. (**A**) Amino acid sequences of zebrafish and a few mammalian TEKT3/Tekt3 are aligned, and the resulted phylogenetic tree (**B**) reveals that zebrafish Tekt3 is older than human TEKT3 in evolution. Zebrafish (*Danio rerio*) is highlighted in red color in (**A**,**B**).

**Figure 2:**
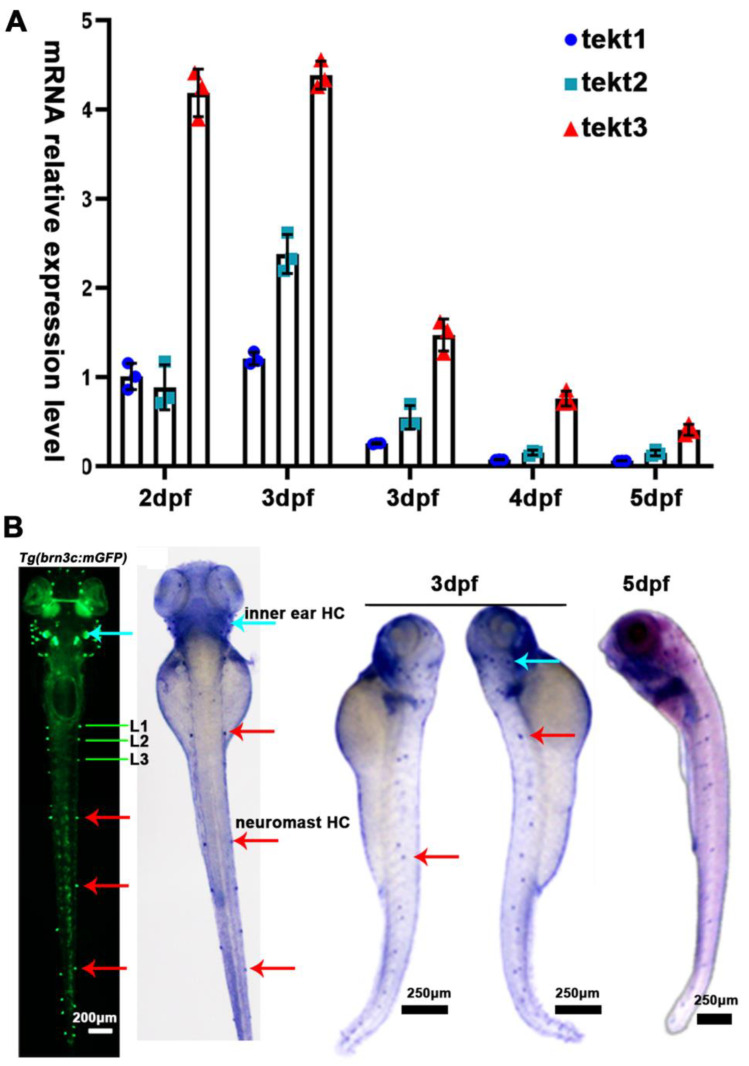
*tekt3* mRNA is specifically present in zebrafish HC-bearing organs. (**A**) The expression profiles of zebrafish *tekt1-3* between 2 and 6 dpf. (**B**) Expression patterns of *tekt3* were detected by the whole-mount in situ hybridization at 3 and 5 dpf. Red arrows point to neuromasts and light blue arrows point to the ear. HC: the hair cell.

Using Neighbor-Joining method, we compared the amino acid sequences of zebrafish and mammalian tektin3-coding genes to indicate the conservation of tekt3 (Figure 1A,B). Whole body qPCR analyses revealed that the *tekt3* mRNA level was much higher than tekt1 and *tekt2* mRNA between 2 and 6 dpf (Figure 2A). Whole-mount in situ hybridization revealed that *tekt3* was exclusively expressed in the inner ear and lateral line neuromast at 3 and 5 dpf (Figure 2B). By analyzing the available single-cell RNA sequencing data [21], *tekt3* mRNA was indeed found to be only expressed in mature neuromast HCs, while *tekt1* and *tekt2* were less abundant (Figure S1).

**Figure 3:**
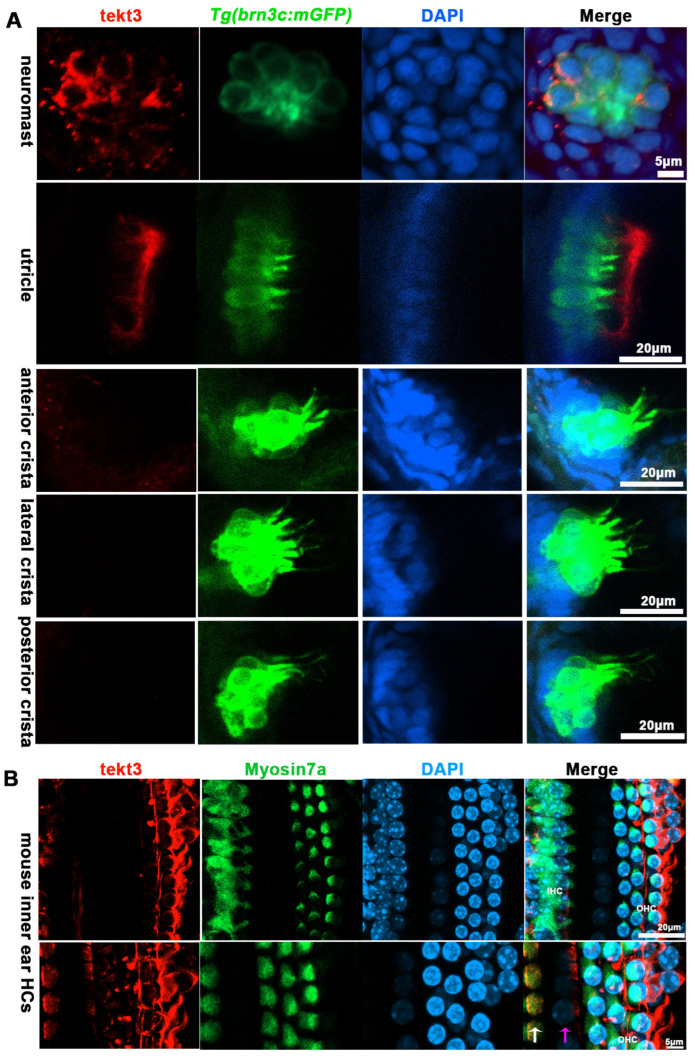
TEKT3/Tekt3 is present in some HCs. (**A**) Neuromast and utricular HCs pf zebrafish larvae at 5 dpf are positive for anti-Tekt3 antibody staining while the crista HCs are negative. (**B**) TEKT3 is present in mature mouse cochlear HCs. The left row of cells are inner HCs (IHC, pointed by a white arrow) and the right three rows are outer HCs (pillar cell, pointed by a pink arrow). Note: The saccular and crista HCs are always negative.

**Figure 4:**
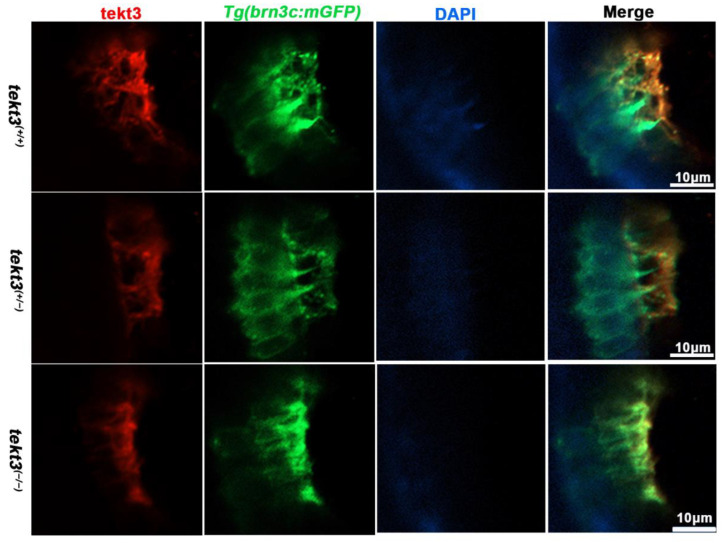
The subcellular location of Tekt3 in zebrafish utricular HCs. Crossed with *Tg*(*brn3c*:mGFP), both homozygous and heterozygous *tekt3* mutants showed weaker α-Tektin3 staining at the utricle (apical portion of HCs). Note: two colors overlap at the very tip portion of hair bundles, yet at the root portion, they never overlap.

Using a tektin3-specific antibody, the immunostaining showed that tekt3 was present in the cytosol of neuromast HCs and the main signal was enriched apically in utricular HCs at 5 dpf (Figure 3A). Interestingly, a barrier plate-like structure, presumably the otolithic membrane (OTM) of the zebrafish’s inner ear, was intensively stained. Furthermore, the OTM-like structure staining did not overlap with the kinocilium-GFP cells but did with some cytosol GFP signals, suggesting the absence of tektin3 in utricular kinocilia and stereocilia. When the utricle HC image was blown up, the cytosolic co-localization of both green and red signals was clearly evident at the apical surface (Figure 4). In mouse cochlea, tektin3 was present in the cytosol and apically enriched in outer HCs (Figure 3B), indicating the conserved subcellular localization. Tektin3 was absent in saccular and crista HCs (Figure 3A). Therefore, *tekt3* expression is quite specific at the young larval stage of zebrafish and tekt3 is mainly present in the cytosol of HCs, and could therefore be used to distinguish sub-types of HCs in both zebrafish and mice.

### 2.2. Disruption of Tekt3 Triggers Genetic Compensation to Boost Total Tektins

**Figure 5:**
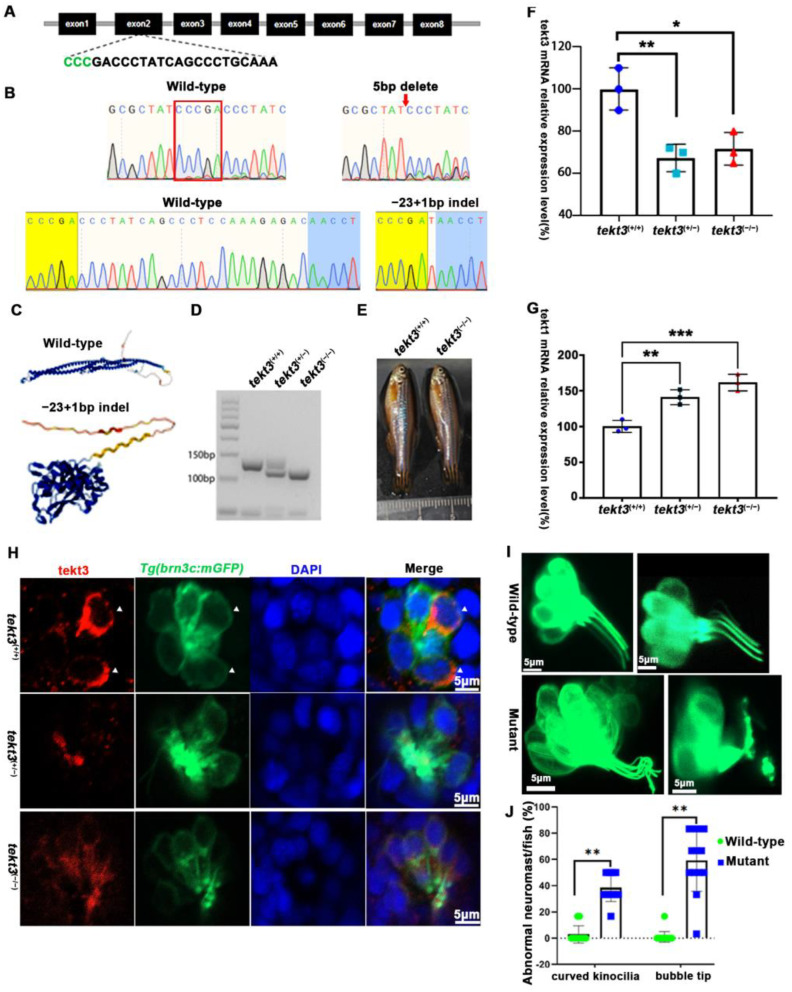
*tekt3* mutant exhibited only neuromast HC kinocilium defect. (**A**) The CRISPR/Cas9-mediated genome editing targeted the exon 2 of *tekt3* locus; the gRNA core sequence is shown. (**B**) Two *tekt3* mutant alleles were verified by sequencing. (**C**) The (Alphfold2.0) predicted protein structure of one mutated Tekt3 is shown side by side with a normal protein. (**D**) A typical genotyping result of *tekt3* mutant. (**E**) A 6 mpf homozygous *tekt3* mutant looked normal. (**F**) Significant reduction in *tekt3* mRNA level can be seen in both homozygous and heterozygous *tekt3* mutants. (**G**) The *tekt1* mRNA level in 5 dpf fish were significantly up in both homozygous and heterozygous *tekt3* mutants. (**H**) Tekt3 was decreased in neuromast HCs of both homozygous and heterozygous *tekt3* mutants (5 dpf). The arrowheads are pointed to tekt3 positive cells. (**I**) The abnormal neuromast HC hair bundle or kinocilia of the 5 dpf *tekt3* mutant. (**J**) Almost all mutant neuromasts (n = 78) showed abnormal kinoclium tips while wild-type (n = 112) rarely possessed the defect. * *p* < 0.05; ** *p* < 0.01; and *** *p* < 0.001.

To better understand the role of tektin3, two *tekt3* mutant lines were obtained (Figure 5A,B). One (indel, +1–23 bp) was most likely a loss-of-function mutant allele (Figure 5C) while the other one (indel, −5 bp) was not. *tekt3* mutant (+1−23) could be easily genotyped via agarose gel electrophoresis (Figure 5D). And the homozygous mutant was vital and fertile (Figure 5E). By qPCR analyses, *tekt1* was found to be upregulated in both heterozygous and homozygous mutants, largely due to some genetic compensation mechanisms, whereas *tekt3* expression was drastically reduced (Figure 5F,G). The homozygous mutant possessed stronger genetic compensation than that of heterozygous mutant. Genetic compensation is crucial for the genetic robustness and could be achieved by functionally redundant genes [22]. The elevation of *tekt1* expression might contribute to the normal fertility of the *tekt3* mutant.

To learn if the mutant is truly a loss-of-function allele, the *tekt3* mutant was crossed into transgenic *Tg*(*brn3c*:mGFP) fish, in which both otic and neuromast HCs can be visualized under a fluorescence microscope. No obvious defect was evident in saccular and crista HCs (Figure S2). In contrast, utricular HCs of the *tekt3* mutant lacked distinct knicilium structure and lower Tekt3 level (Figure 4). Mutant neuromast HCs also produced much less Tekt3 and their kinocilia were curved/bent with or without a bubbled tip (Figure 5H,I). To quantify the mutant phenotype, we counted the number of abnormal neuromasts (L1–L3/fish) at 5 dpf. Among 17 wild-type fish and 112 neuromasts, only two neuromasts showed slightly bent hair bundles and one carried a tiny bubble at the tip of the kinocilia (Figure S3). The curved kinocilium tips, kinocilia with bubbled tips, and hair bundles mixed with both types of kinocilia were almost exclusively present in 78 *tekt3* mutant neuromasts (Figure 5J). Moreover, Tekt3 was largely enriched apically in neuromast HCs (Figure 5H).

### 2.3. A Lack of Tekt3 Leads to Neuromast Function Defects

**Figure 6:**
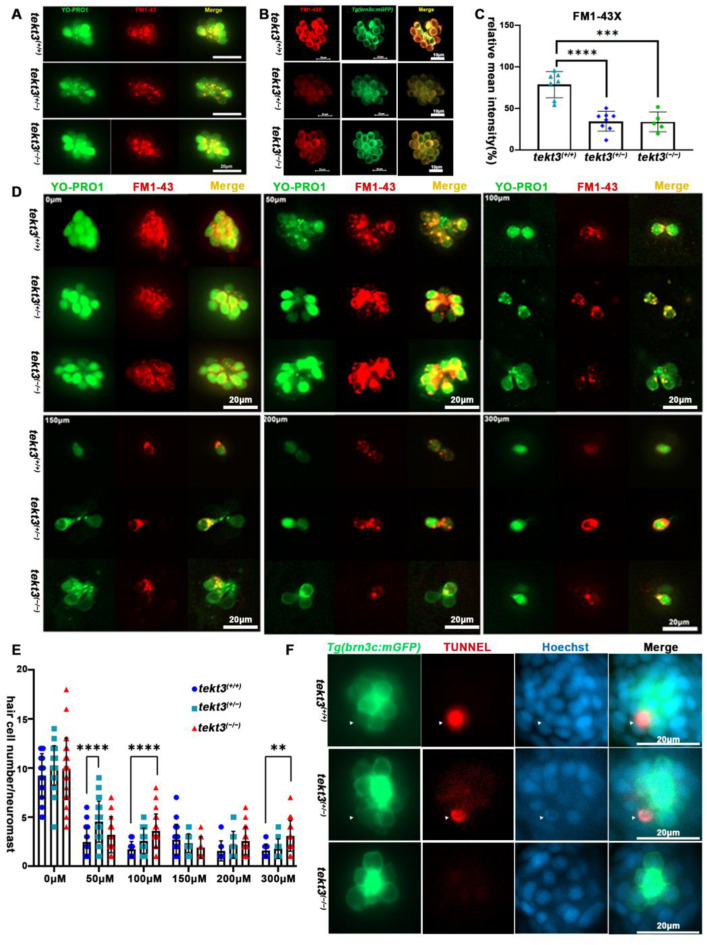
Neuromast HCs of *tekt3* mutant are neomycin resistant. (**A**) Live dye (YO-PRO1 and FM1-43) intake by neuromast HCs in 5 dpf WT and *tekt3* mutant. (**B**) YO-PRO1 and FM1-43X staining. (**C**) Weaker dye-intaking of *tekt3* mutant neuromast. (**D**) Resistance to neomycin treatment. (**E**) Neomycin dose-dependency. (**F**) Weaker TUNNEL signals of mutant neuromast after neomycin treatment. The arrowheads are pointed to apoptotic neuromast hair cells. ** *p* < 0.01; *** *p* < 0.001; and **** *p* < 0.0001.

To distinguish if the morphological defect of HC kinocilia is due to developmental defect or function-triggered collapse of kinocilia upon the lack of Tekt3, we first assessed HC functions. From YO-PRO1 and FM1-43 or FM1-43X staining, the heterozygous mutant showed a significantly slower intake of the vital dyes (Figure 6A–C), indicating a possible MET blockage in neuromast HCs. To verify the possibility, neomycin was used to kill pre-existing neuromast HCs for 0.5 h, and the remaining neuromast HCs were counted, revealing that the *tekt3* mutant is indeed partially resistant to neomycin-induced HC death (Figure 6D,E). The lower TUNNEL signal was evident in both homozygous and heterozygous *tekt3* mutant neuromasts, likely supporting a Tekt3-enabled protective effect on HCs upon a 5 min treatment of 300 μM neomycin (Figure 6F).

**Figure 7:**
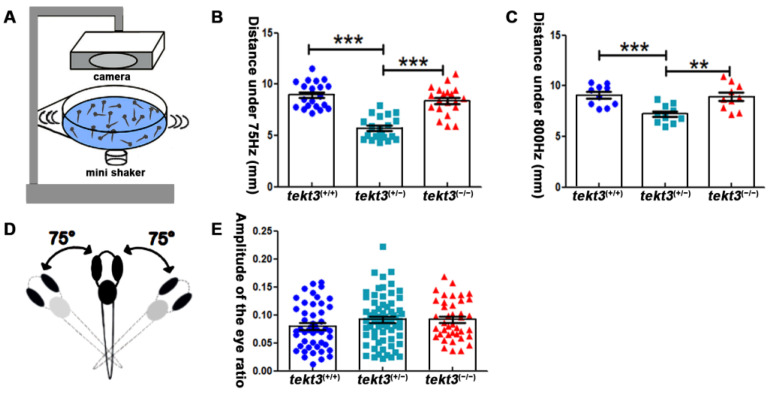
Heterozygous *tekt3* mutant has weaker response to soundwave stimuli. (**A**) Self-made equipment to measure neuromast function (**B**,**C**) showed that the 6 dpf heterozygous *tekt3* mutant exerted weaker response upon 75 Hz (**B**) or 800 Hz (**C**) stimuli. (**D**) A diagram of a larval zebrafish in linear VOR test. (**E**) The 5 dpf *tekt3* mutant had a normal VOR score. ** *p* < 0.01; and *** *p* < 0.001.

Zebrafish neuromasts can partially mediate vibrational stimuli triggered startle response that can be captured by a self-made equipment (Figure 7A). By measuring the moving distance upon 75 Hz and 800 Hz stimuli (Figure 7B,C), the heterozygous *tekt3* mutant was more affected, perhaps also due to the genetic compensation via elevated *tekt1* expression. However, the linear VOR test showed no obvious defect of the *tekt3* mutant utricle (Figure 7D,E). These results indicated that only neuromast function was negatively affected in the absence of Tekt3, although *tekt3* is expressed in both utricle and neuromast HCs. These experiments suggest a critical role of Tekt3 to safeguard functional integrity rather than to affect HC development.

### 2.4. Lacking Tektin3 Delays HC Regeneration of Neuromast After Neomycin Treatment

**Figure 8:**
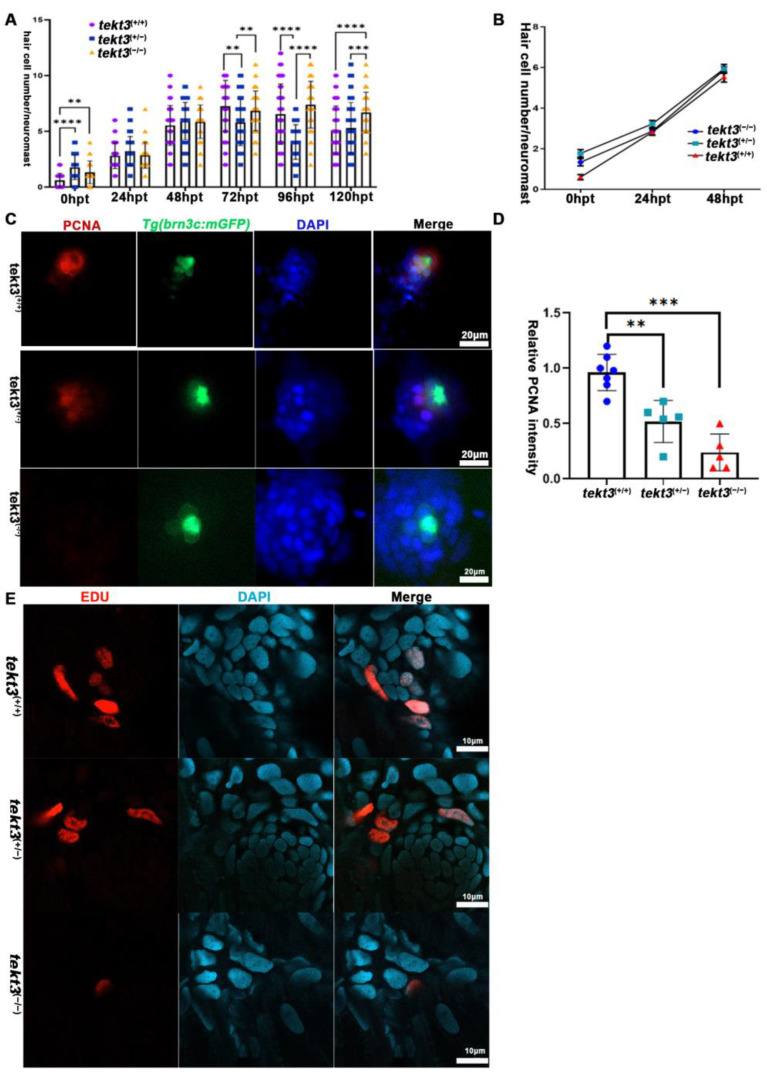
Neuromast HC regeneration is delayed in *tekt3* mutant. (**A**) The summary of HC numbers in L1-L3 neuromasts, days after neomycin administration. (**B**) The HC regeneration. (**C**,**D**) PCNA staining shows neuromast cell proliferation defect of *tekt3* mutant. (**E**) EDU staining shows neuromast cell proliferation defect of *tekt3* mutant 6 h after neomycin treatment. ** *p* < 0.01; *** *p* < 0.001; and **** *p* < 0.0001.

Upon neomycin treatment to kill pre-existing HCs, neuromast HC regeneration program is triggered. If neomycin intake is slower in *tekt3* mutant HCs, neuromast HC regeneration, in theory, should be slower than that of wild-type neuromast. We treated the larvae with 300 μM neomycin at RT for 30 min and showed that *tekt3* mutant was indeed late in regenerating HCs (Figure 8A,B). This negative effect is due to slower cell proliferation of non-HCs, as demonstrated by PCNA and EDU staining experiments (Figure 8C–E).

**Figure 9:**
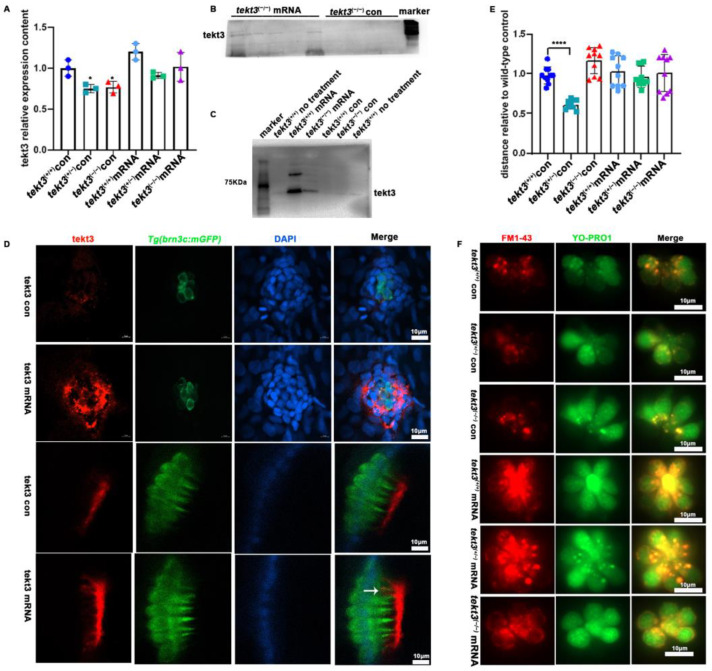
The rescue of *tekt3* mutant. (**A**) Comparison of *tekt3* mRNA levels between control and experiment groups is shown. (**B**,**C**) The protein level could be brought back a bit in the injected groups. (**D**) Rescued neuromast and utricle of *tekt3* mutant. (**E**) The rescued startle response in mRNA injection groups. (**F**) The YO-PRO1 and FM1-43 intakes become normal in mRNA injected groups. The white arrow is pointed to a kinocilium with positive tekt3 signals. * *p* < 0.05; and **** *p* < 0.0001.

To ultimately ascertain how tektin3 is related to the phenotypes observed above, we constructed a *tekt3*-expressing vector by inserting the full-length CDS of wild-type *tekt3* into pblk-CMV-SV40. The wild-type and mutant fish, co-injected with TP mRNA and pblk-CMV-*tekt3*-CDS-SV40 vector, were evaluated by PCR, antibody staining and Western blot, and successful rescues were scored (Figure 9A–D). The rescued mutant fish showed better response to the soundwave stimuli (Figure 9E) and improved uptake of YO-PRO1 and FM1-43 (Figure 9F). Interestingly, the presence of Tekt3 was observed in a few kinocilia of the injected wild-type fish (Figure 9D). The fact that the *tekt3* mutant possesses functional defects in its neuromasts, which were largely rescued by mis-expressing wild-type *tekt3*, indicated that Tekt3 is responsible for the mutant phenotypes.

## 3. Discussion

The present study was initially designed to provide supporting evidence of a hypothesis that microtubule-associated Tekt3 is a component of HCs to stabilize structure and safeguard function of kinocilia. After checking the specificity of *Tekt3*/*tekt3* expression in zebrafish larvae, we have discovered that Tekt3/*tekt3* is only present in utricular and neuromast HCs; it is absent in HCs of saccule and ampulla crista. We have additionally unveiled that Tekt3 is largely present in the cytosol of neuromast and utricular HCs in zebrafish, and TEKT3 is found in the cytosol of cochlear outer HCs in mature mice. We then bred a loss-of-function *tekt3* mutant that develops normally and is fertile, i.e., no obvious sperm defect.

All HCs of the zebrafish inner ear and lateral line possess kinocilium-containing hair bundles. Since tekt3 is not present in all HCs of these organs, one may predict that zebrafish tekt3 is not an essential kinocilium component, regarding the structure and/or function. Given that kinocilia should act similarly in mechanoelectrical transition, Tekt3 may play a specific, balance-related role unique to utricular and neuromast HCs. To make the situation more complex, however, the loss-of-*tekt3*-function mutant has morphological and function defects of kinocilia only in neuromast HCs.

It is possible that the subcellular location of Tekt3 matters. In addition to the apical cytosol-enriched Tekt3, the immunochemical signal is also present in OTM at the utricle. OTM is composed of a dozen proteins and helps the tips of HC hair bundles to avoid direct contact with the otolith and mediates the movement of otoliths in response to soundwave/vibration triggered mechanical stimulation. Occasionally, Tekt3 was detected in a mesh-like structure at the top of neuromast hair bundles (Figure S4). In a 5–6 dpf neuromast, HCs stick out their apical portion of kinocilia that are embedded in the cupula. After PFA fixation, ~30% of the sampled cupula adopted a mesh-like structure (unpublished observation). The fact that both utricular OTM and neuromast cupula are completely outside the cell body of HCs suggests that Tekt3 might be present at the tip portion of a kinocilium in these HCs. The compartmentation of primary cilia, including zebrafish crista, and neuromast kinocilia, along the proximal-distal axis, could be visualized using labeled/fusion kinocilium-specific proteins [2,23,24]. It is thus possible that PFA fixation breaks kinocilia to allow OTM to retain tip-located Tekt3. The jelly cupula of neuromast, however, may not be able to firmly retain Tekt3 during PFA fixation. It is also possible that kinocilia are extremely fragile [25] and PFA fixation helps to quick lock Tekt3 at kinocilium tip. If so, the strong intensity of Tekt3 in OTM, much higher than that in neuromast cupula, means a higher Tekt3 level in utricular HCs. Nevertheless, live visualizing Tekt3 would ultimately help to resolve the question of whether Tekt3 is truly located in the kinocilium tip.

Alternatively, if Tekt3 is not permanently located in neuromast and utricular kinocilia, the large amount of Tekt3 present in OTM may suggest an active transportation of Tekt3 intracellularly in utricular HCs. When *tekt3* is mis-expressed, few kinocilia are tekt3^+^, also suggesting a traffic of Tekt3 between kinocilia and cytosol of utricular HCs. Perhaps Tekt3 is actively transported in neuromast HCs too (Figure S4) if appropriate fixation is employed. Thus, what type(s) of subcellular event involves Tekt3 may warrant further investigation. As a hint for future research, mammalian TEKTs are found to be not restricted to microtubule-associated structures. In rat spermatozoa, TEKT3 is not directly associated with the flagellar axoneme. Instead, TEKT3 is a component of the peri-axonemal complex and predominantly associated with the surface of mitochondria [16]. But what exactly TEKT3 does in these locations remains unknown.

Zebrafish neuromast HCs are constantly challenged by various low frequency mechanical and vibrational stimulation in a laboratory setting, thus requiring constant recharge or repair. That Neuromast HCs are more active in terms of metabolism than inner ear HCs [11,26] appears to support such a notion. While the chemical composition of neuromast cupula remains unidentified, its protective strength may be much weaker than that of inner ear OTM. If so, less protected neuromast HCs need a quick turnover of Tekt3 for some reason. Otherwise, the mutant kinocilia damage shows up, exhibiting the bent or bulbed kinocilium tips. A better protection mediated by OTM may account for normal HC structure and function of mutant utricular HCs.

The genetic compensation caused by enhanced *tket1* expression in the *tekt3* mutant is evident (Figure 5G). However, defects of vital dye intake and vibration-triggered startle response are only evidenced in the heterozygous mutant (Figure 6C and Figure 7), suggesting that elevated *tekt1* can partially replace the role of *tekt3* in fine-tuning neuromast function in homozygous mutants. In contrast, elevated *tekt4* expression fails to compensate *tekt3* at all in the mutant (unpublished observation). Nonetheless, injected normal *tekt3* mRNA can rescue mutant abnormalities, indicating that Tek3 is hard to be replaced and may help to achieve kinocilia diversity during the evolution of tektin family in teleost.

In parallel, we observed that mouse TEKT3 is apically enriched in the cytosol of outer HCs of mature mouse cochlea but is absent in cilia (Figure 3B). Therefore, HCs of mouse cochlea and zebrafish utricle express *Tekt3*/*tekt3* and distribution of TEKT3/Tekt3 exerts somewhat similar subcellular distribution. However, zebrafish saccular HCs and mouse cochlear inner HCs, the true sound sensor cells in both animals, both lack the protein, suggesting less relevance of TEKT3 to hearing loss disease of humans. Defects in the sensory function or structure of primary cilia are known to be associated with diseases termed ciliopathies, and hearing loss (HL) in relation to HCs’ ciliary defects is also considered to be one such kind. Studies on cilia-related HL have largely been focused on stereocilia, since mature cochlear HCs lack any kinocilia [27]. Cochlear stereocilia are responsible for the conversion of mechanical energy into electrical signals in mammalian ears. Kinocilia have drawn a growing interest due to their important roles in the development of hair bundle polarity and the maintenance of normal hearing. For instance, the loss of kinociliary links leads to abnormal polarity of hair bundles. Genetic defects result in the absence of cilia, morphologically defective cilia, or abnormally elongated cilia, which result in hearing loss in mice [28,29,30,31]. The present study suggests that *TEKT3* can be listed as a risk factor of noise-triggered and cochlear outer HC-related hearing loss/deafness in humans.

## 4. Materials and Methods

### 4.1. Zebrafish Husbandry and Care

The wild-type AB strain and transgenic zebrafish line *Tg*(*Brn3c:mGFP*) were raised as previously described [32]. Briefly, all strains were maintained at 28 °C and a density of about 10 fishes/L under a 14 h light/10 h dark cycle. Embryos and larvae were raised in the E3 medium under 28 °C conditions. All animal experiments were approved by the Institutional Animal Care and Use Committee of Southern University of Science and Technology.

### 4.2. Quantitative Real-Time PCR

Total RNA was extracted from 30 wild-type or mutant larvae at 5 dpf using TRIzol reagent (Invitrogen, Waltham, MA, USA). To be more specific, 1 mL TRIzol was added into the larvae and ground with a TGrinder (TIANGEN, Beijing, China, OSE-Y30) for about 2 min. Then, 200 μL chloroform was added and mixed by vigorously shaking the tubes for 30 s and they were then incubated for 5 min at RT. After centrifuging, the upper 200 μL aqueous phase was carefully transferred into a fresh tube. Then, the same volume of isopropanol was added and gently mixed by inverting the tube. Then, they were incubated at RT for 5 min. After centrifuging, the RNA was washed with 75% cold ethanol. The RNA pellet was air-dried at RT and dissolved in nuclease-free water. The concentration and quality were measured with a Nanodrop and Agilent 2100 bioanalyzer (Thermo Fisher Scientific, Waltham, MA, USA). A total of 1μg RNA was reverse transcribed to the first strand of complementary DNA with the random primer using a complementary DNA synthesis kit (Promega, Madison, WI, USA, A5003). Quantitative real-time PCR primers for *tekt* gene members and *gapdh* are listed in Table S1. The real-time PCR reaction was set up with a SYBR^®^ qPCR Master Mix (Vazyme, Nanjing, China). The PCR was performed in an ABI 7500 Real-Time PCR instrument (Applied Biosystems, Waltham, MA, USA) with the SYBR green detection system, and results were normalized with *gapdh* expression using ^ΔΔ^Ct method.

### 4.3. Neomycin Treatment

The 5 dpf embryos were rinsed in culture medium containing different concentrations of neomycin (Merck Millipore, Burlington, MA, USA, 4801-25GM) at 28 °C for 30 min, then the embryos were gently washed with culture medium without neomycin 3 times and used for other detections.

### 4.4. Immunofluorescence Staining

For immunofluorescence, the zebrafish larvae were maintained with 0.2 mM PTU (1-phenyl-2-thiourea, Sigma, Saint Louis, MO, USA) solution after 24 hpf. Then, the 5 dpf zebrafish larvae were anesthetized with 0.016% (*w*/*v*) anesthetic tricaine/E2 medium (MS-222, pH = 7.0, Sigma, Shanghai, China, E10521) on ice for 3 min. After removing most of the fluid, the larvae were fixed with 4% paraformaldehyde (PFA)/PBS at 4 °C overnight. After three washes with 0.2% Tween-20/PBS, the fixed larvae were incubated with 0.1% Triton X-100/PBS at RT for 30 min. Then, the larvae were blocked in a blocking buffer containing 5% donkey serum (*v*/*v*), 3% BSA (*w*/*v*), and 0.1% Trixon X-100 (*v*/*v*) at RT for 1 h. The larvae were incubated with diluted primary antibody at 4 °C overnight. After three times of PBST wash, each for 5 min, the larvae were incubated with diluted secondary antibody at RT in dark for 2 h. Then, larvae were washed and stained with a 1:1000 dilution of DAPI in dark for 5 min. Finally, the larvae were imaged with a Zeiss LSM780 confocal microscope (Oberkochen, Germany) using 20× object lens.

The wild-type C57BL/6 mice were transcardially perfused with 4% (*w*/*v*) PFA/PBS after anesthesia with isoflurane. The cochlea was collected and fixed in 4% PFA at 4 °C overnight and then decalcified with JYBL-I decalcifying fluid (Solarbio, Beijing, China, G2470) at 4 °C for 2 days. Then, the tissues around HCs were cut off and the HCs were used for staining. Similarly, the tissues were penetrated with 0.1% Triton X-100/PBS at RT for 30 min, blocked with 5% donkey serum (*v*/*v*), 3% BSA (*w*/*v*), and 0.1% Trixon X-100 (*v*/*v*)/PBS at RT for 1 h. Then, the HCs were incubated with primary antibodies at 4 °C overnight and secondary antibodies at RT in dark for 2 h. Finally, the HCs were mounted with DAPI Fluoromount-GTM mounting medium (20 μL/slice, Yeasen, Shanghai, China, 36308ES11) and imaged with a Zeiss LSM780 confocal microscope using Z-stack projections.

The primary antibodies used were rabbit anti-Tekt3 (1:100, Proteintech, Rosemont, IL, USA, 12959-1-AP-50UL), anti-PCNA (1:100, Proteintech, 10205-2-AP-50UL), and Anti-MYO7A (1:50, DSHB, Iowa, USA, 138-1). The secondary antibodies used are donkey anti-rabbit Alexa Fluor-568 (1:1000, Invitrogen, A10042) and donkey anti-mouse Alexa Fluor-488 (1:1000, Invitrogen, A21206).

As for FM™1-43FX staining, FM™1-43FX dye (300 μM, Thermo Fisher, F35355) was used for incubating the living embryos for 15 s in dark at RT. Then, the embryos were fixed in 4% PFA at RT for 15 min, and the embryos were imaged with a Zeiss LSM780 confocal microscope.

### 4.5. FM1-43 Intake Assay and YO-PRO1 Staining

The fluorescent vital dye FM1-43 can rapidly and specifically label active hair cells by entering through open mechanotransduction channels [33,34]. The intake of FM 1-43 is commonly used as a hair cell mechanotransduction marker in many animal models, including zebrafish [35]. YO-PRO-1, a cyanine dye, which can entry into hair cells through non-selective mechanotransduction channels and bind to the DNA, is also a reliable indicator of hair cell viability and has been used in hair cell studies [35]. To recognize the functional HCs in neuromasts, a FM1-43 staining experiment was conducted as follows. The free-swimming larvae were incubated in 3μM FM1-43 vital dye (Thermo Fisher, T3163) for 25 s at room temperature (RT) in the dark. Then, the larvae were gently washed with E2 buffer twice. Similarly, 200 μM YO-PRO1 (Thermo Scientific, Y3601-4) was stained in dark at RT for 1 min and washed with culture medium. Finally, the larvae were imaged after being gently mounted in 1% low melted agarose containing 0.016% (*w*/*v*) anesthetic tricaine.

### 4.6. Whole-Mount in Situ Hybridization

The whole-mount in situ hybridization (WISH) of zebrafish was performed according to the following standard procedures. The cDNA fragment of zebrafish tekt3 was amplified via PCR using designed primers—tekt3-ISH-F, 5′AGATTTCAGCGCTGTCCGAT′ and tekt3-ISH-R 5′ AAGCAGCACGTTCACTCTGA3′—and was cloned into an EZ-T cloning vector with an EZ-T Fast Ligation Kit (GenStar, #T168-10, Beijing, China). After linearization of the EZ-T-inserting tekt3 fragment, the DIG RNA Labeling Kit (T3 & T7) (Roche, #11175025910, Indianapolis, IL, USA) was used to prepare digoxigenin-labeled tekt3 sense and antisense mRNA probes through transcription in vitro. The 3 dpf and 5 dpf embryos were anesthetized with 0.016% (*w*/*v*) anesthetic tricaine/E2 medium on ice for 3 min and then fixed in 4%PFA at 4 °C overnight, digested in proteinase K at RT for 10 min, and incubated with a pre-hybridized mix at RT for 1 h. After that, the embryos were hybridized with tekt3 sense or antisense probes overnight at 65 °C. After incubation with alkaline phosphatase (AP)-conjugated antibody against digoxigenin (Roche, #11093274910) at 4 °C overnight, the NBT/BCIP solution (Roche, #11681451001) was used to detect the tekt3 mRNA expression.

### 4.7. CRISPR/Cas9 Generated tekt3 Mutant

The tekt3 mutant zebrafish were generated via CRISPR/Cas9-mediated gene editing technology. The single guide RNA (sgRNA), specifically targeting exon 2 of tekt3 (5′-TTT GCA GGG CTG ATA GGG TC-3′), was synthesized (Genescript, Nanjing, China). Then, the sgRNA was dissolved in nuclease-free water. A mixture of 300 ng/μL Cas9 protein (NEB, Ipswich, MA, USA, M0646T) and 100 ng/μL *tekt3* sgRNA was co-injected into one-cell stage zebrafish embryos. Then, those injected embryos (designated as F0) were raised and outcrossed with wild-type zebrafish to achieve F1 fish. F1 fish were raised to adulthood. A small piece of caudal fin of these adult F1 fish was cut and used for genotyping. The DNA was extracted with 30μl 50 mM NaOH at 97 °C for 30 min and adjusted the pH with 1 mM Tris-HCl (pH 7.2). The genotype of F1 fish was first examined with PCR. Then, the genotype of the F1 fish was further identified with Sanger sequencing. Finally, the heterozygous F1 fish were kept and incrossed to obtain a stable *tekt3* mutant line. A pair of primers, at both ends of the *tekt3* target site, were designed and synthesized for genotyping. The used genotyping primers were: tekt3-gF: 5′-CAGAACTCCAATATGCCCTGG-3′ and tekt3-gR: 5′-CTGTCCTGTTCGAGGGGTAGA-3′.

### 4.8. Vestibulo-Ocular Reflex (VOR) Assay

A customized VOR testing system was obtained from the Southern University of Science and Technology and used to quantify linear VOR in zebrafish larva, evoked by the head motion to the earth horizontal axis. The detailed procedure for linear VOR testing are as follows. The zebrafish larva was gently mounted in the larva-shaped chamber in a dorsal-up position with the tail glued by 5% methylcellulose and covered with a piece of glass coverslip on the chamber. After adding E3 embryo media in the head region, the chamber unit was then mounted on a device for quantifying linear VOR. After aligning the larval eyes to the center of the infrared camera, the platform started to rotate back and forth around a horizontal axis at a speed of 30 rpm, and the VOR was recorded by the camera [36].

### 4.9. Startle Response Assay

The sensory function of zebrafish was tested by the startle response assay. In brief, a plastic plate attached to a mini vibrator was used to place 20 normal larvae at 6 dpf, while an infrared digital video tracking system was used to monitor their swimming behavior. The zebrafish lateral line can sense low-frequency stimulation up to 200 Hz, and the inner ear can detect stimulation higher than 100 Hz [37,38]. For indirect stimulation, the zebrafish frequency response curve studies show that best response frequency of lateral line distributes at 74 ± 28 Hz [39]. After removing neuromasts, the hearing is not affected at about 800 Hz in zebrafish [40]. In order to detect the sensory functions of both neuromasts and ear, the 75 or 800 Hz with 32 dB re.1 ms^−2^ tone bursts were applied to the amplifier to drive the vibrator. The conditions of acoustic vibration stimuli lasting for 30 ms with a 180 s inter-stimulus interval were set and applied. Each sound vibration stimulus level was repeated for 20 times and the locomotion behavior of the larvae with C-shape motion to this stimulus was recorded. Finally, the movement typical parameters of mean distance and peak velocity were analyzed to assess the startle response of larvae to sound vibration stimuli.

### 4.10. Western Blot Analysis

The 5 dpf zebrafish were homogenized in a cold RIPA lysis buffer (GLPBIO, Shanghai, China) with a protease inhibitor cocktail (CST, Danvers, MA, USA, #5871S). Protein concentration was determined using the BCA protein assay kit (Biomed, Guangzhou, China). Proteins were separated with 10% SDS-PAGE (EpiZyme, Shanghai, China, PG112 ) and transferred to PVDF membranes (Beyotime, FFP39, Shanghai, China). The membranes were blocked with 5% skimmed milk dissolved in TBST buffer at RT for 1 h and then incubated with the Anti-TEKT3 antibody (1:1000, Proteintech) and Anti-GAPDH (1:000, SAB) at 4 °C overnight with gentle shaking. After washing in 1×TBST buffer (20 mM Tris–HCl, 150 mM NaCl, 0.05% Tween 20, and pH 7.6) 3 times, the membranes were incubated with HRP-conjugated secondary antibodies (1:1000; Beyotime) for 2 h at RT. The membranes were then developed using Tanon^TM^ High-sig ECL Western Blotting Substrate (Tanon, Shanghai, China, 130-301) and Tanon6100C imaging system (Tanon). Quantitative analysis of protein bands was performed by the Tanon 6100C software.

### 4.11. Construction of pblk-CMV-tekt3-CDS-SV40 Vector and Microinjection

A vector containing full-length *tekt3* CDS was constructed. The full-length *tekt3* CDS was amplified from 6 dpf wild-type AB strain cDNA with the following primers: *tekt3*-csdF: 5′-CCATCGATTCGAATTCAAGGATGGAGCTCATCGGATCCACCC-3′ and *tekt3*-csdR: 5′-CTTATCATGTCTGGATCTACCTATGTAAATCCAACCAGGCGA-3′. Then, the PCR product was inserted into a pblk-CMV-SV40 vector via a Gibson Assembly HiFi Kit (Invitrogen, A46624EA). The vector was examined by Sanger sequencing. Then, 50 ng/μL vector and 100 ng/μL TP mRNA were co-injected into one-cell stage embryos.

### 4.12. TUNEL Assay

TUNEL staining was conducted according to the manufacturer’s instructions of the One Step TUNEL Apoptosis Assay Kit (Beyotime, C1089, Shanghai, China). In brief, embryos were fixed in 4% PFA at RT for 30 min and permeated with 0.3% Triton X-100/PBS at RT for 5 min, then incubated with the TUNEL-detected buffer (10% TdT enzyme, and 90% Cy3-labeled buffer) in the dark at 37 °C for 60 min. Finally, the embryos were washed with PBS and were imaged under a fluorescent microscope (Zeiss, Axio Observer7) using 550 nm excitation and 570 nm emission.

### 4.13. EdU Staining Assay

Hair cell regeneration assessment was conducted utilizing EdU Apollo 567 In Vitro Kit (ACMEC AC10869, Shanghai, China). The experiments were conducted according to the protocol provided by the manufacturer. In brief, 6 dpf larvae were treated with 300 μM neomycin for 30 min and then incubated with 2.5μM EDU-A buffer for 6 h. Then, the larvae were anesthetized with 0.016% (*w*/*v*) anesthetic tricaine and fixed with 4% PFA at RT for 30 min, penetrated with 0.5% Triton X-100 (Coolaber, Shanghai, China) in PBS for 20 min at RT, and then incubated with the 1×straining buffer which contains buffer B-E in the dark at RT for 30 min. Finally, the larvae were washed with 0.5% Triton X-100 in PBS for 15 min and incubated with DAPI for 5 min in the dark. The signal was obtained with a Zeiss LSM780 confocal microscope.

### 4.14. Statistical Analysis

All the experiments were repeated at least three times. The data are presented as mean ± SD. An unpaired two-tailed Student’s *t*-test was used for statistical analysis by GraphPad Prism 8.0 Software. A value of *p* < 0.05 between groups was considered statistically significant.

## 5. Conclusions

In this study, we have discovered that *tekt3* is specifically expressed in both utricular and neuromast HCs, but that a kinocilium defect, i.e., a curved or bubbled cilium tip, was only evident in neuromast HCs of a zebrafish *tekt3* mutant. Moreover, neuromast functions such as the intake of FM1-43 or neomycin and the response to vibrational stimulation were reduced in the *tekt3* mutant. These defects could be rescued by injecting the full-length of normal *tekt3* mRNA into one-cell stage embryos. In addition, the *tekt3* mutant exhibited a lower cell proliferation rate and lower apoptotic signal in neomycin-damaged neuromasts, consistent with a late start of neuromast HC regeneration after neomycin treatment. Our study has demonstrated a specific role of Tekt3 in maintaining the integrity and functions of neuromast HCs in zebrafish.

## Data Availability

The data presented in this study are available in the article and Supplementary Materials.

## Supplementary Materials

The following supporting information can be downloaded at: https://www.mdpi.com/article/10.3390/ijms26073115/s1.
