# Supplementary material for: Tekt3 Safeguards Proper Functions and Morphology of Neuromast Hair Bundles
Source: Int J Mol Sci. 2025 Mar 28;26(7):3115. doi: 10.3390/ijms26073115 (PMC11989051; doi:10.3390/ijms26073115)
Supplement: Supplementary file 1 [file ijms-26-03115-s001.zip › ijms-3415817-supplementary.pdf]

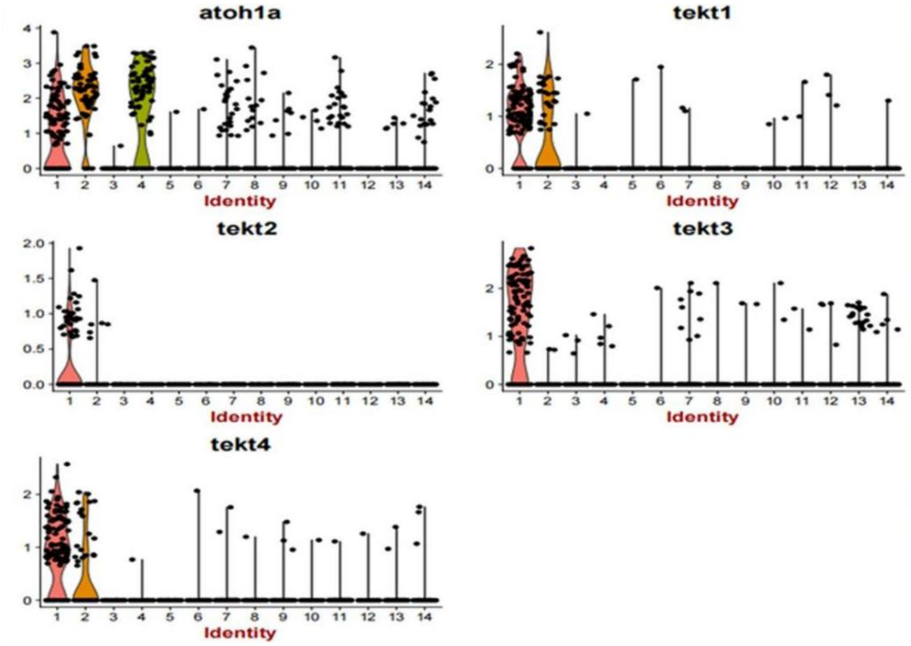

Figure S1. ***tekt3*** **is specifically expressed in the hair cells of zebrafish.** Violin plots show how Tektin family genes and *atoh1a* are expressed/distributed in 14 cell clusters via analyzing the scRNA-Seq data from https://piotrowskilab.shinyapps.io/neuromast_homeostasis_scrnaseq_2018/ (accessed on 8 November 2023) [21]

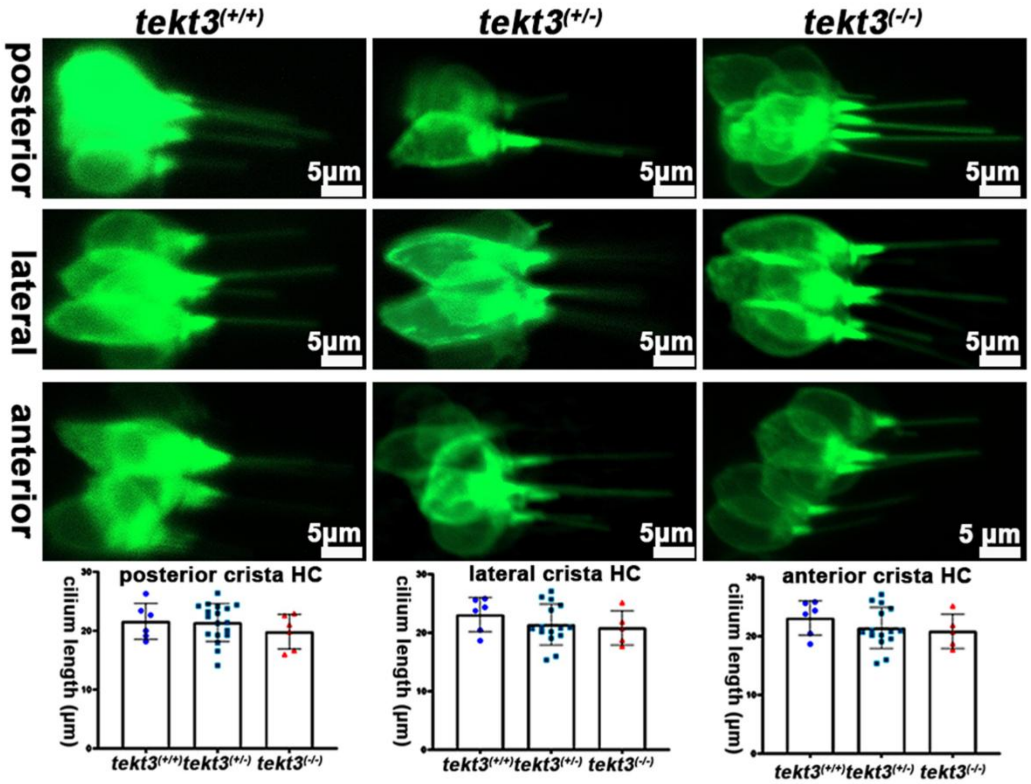

Figure S2. All crista HCs of 5dpf ***tekt3*** **mutant appeared to be normal.** The morphology and averaged kinocilium lengths of each HC are not obviously changed between wild-type and *tekt3* mutant fish. HC: hair cell.

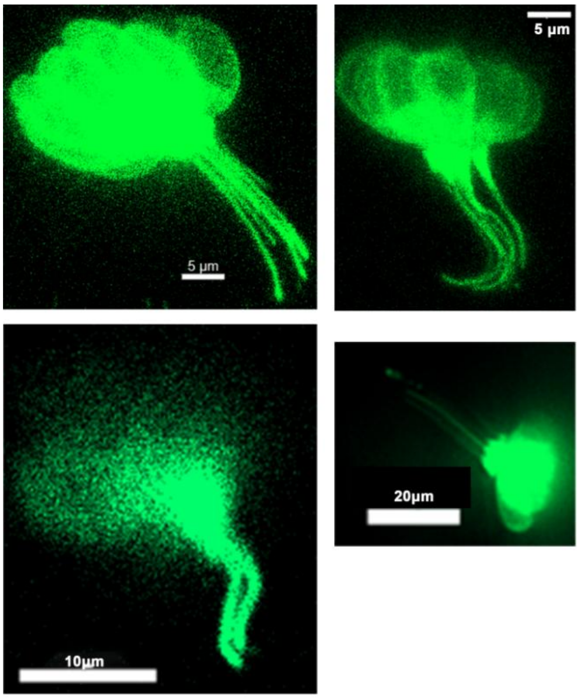

Figure S3. Kinocilium defect of neuromast HCs is rarely detected in 5dpf wild-type fish. Out of 112 WT neuromasts, only three showed minor defect of their hair bundles, compared to the normal looking one at upper left corner.

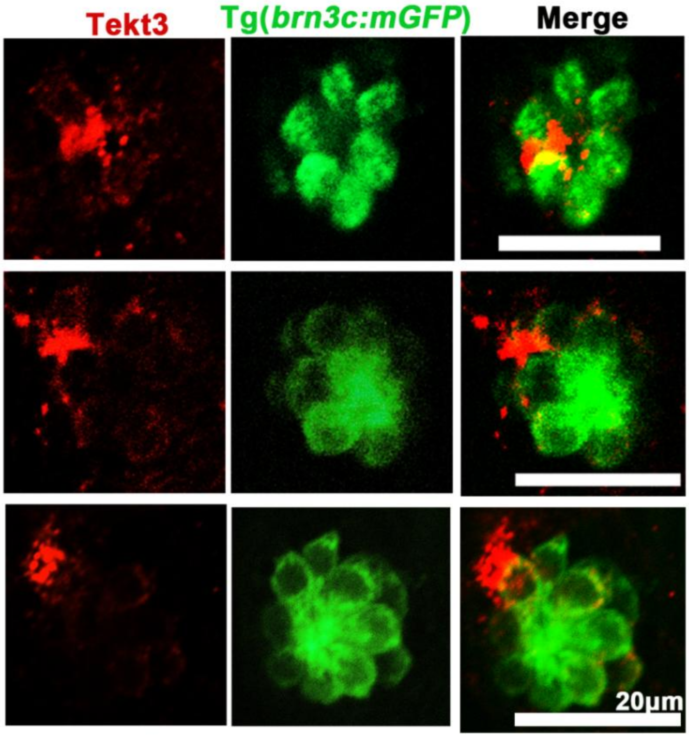

Figure S4. Is Tekt3 present in neuromast cupula? In ~30% fixed neuromasts, a mesh-like structure with Tekt3 signal could be detected. Three examples are shown.

Table S1. The primers used for Q-PCR.

Gene	Forward primer	Reward primer
tekt1	5’-GCAGTACAGAGGCGTTTAG-3’	5’-AGCTTCTGTAATGTTCCTGG-3’
tekt2	5’-ATCAGTCTTCCCGATACTGTG-3’	5’-TTCCCTCAGATTCAAGCACT-3’
tekt3	5’-CAGAACTCCAATATGCCCTGG-3’	5’-CTGTCCTGTTCGAGGGGTAGA-3’
tekt4	5’-CAACCAGATAAGGCTGAACC-3’	5’-GCCAAAATGCACACATACTG-3’
gapdh	5’-ATGCCGAACGGAGACGAG-3’	5’-GCACTCCATCATCATTGCTTTG-3’
